# Standing task difficulty related increase in agonist-agonist and agonist-antagonist common inputs are driven by corticospinal and subcortical inputs respectively

**DOI:** 10.1038/s41598-019-39197-z

**Published:** 2019-02-21

**Authors:** Tulika Nandi, Tibor Hortobágyi, Helco G. van Keeken, George J. Salem, Claudine J. C. Lamoth

**Affiliations:** 1 Center for Human Movement Sciences, University of Groningen, University Medical Center Groningen, Groningen, The Netherlands; 2 Division of Biokinesiology and Physical Therapy, University of Southern California, Los Angeles, CA USA

## Abstract

In standing, coordinated activation of lower extremity muscles can be simplified by common neural inputs to muscles comprising a functional synergy. We examined the effect of task difficulty on common inputs to agonist-agonist (AG-AG) pairs supporting direction specific reciprocal muscle control and agonist-antagonist (AG-ANT) pairs supporting stiffness control. Since excessive stiffness is energetically costly and limits the flexibility of responses to perturbations, compared to AG-ANT, we expected greater AG-AG common inputs and a larger increase with increasing task difficulty. We used coherence analysis to examine common inputs in three frequency ranges which reflect subcortical/spinal (0–5 and 6–15 Hz) and corticospinal inputs (6–15 and 16–40 Hz). Coherence was indeed higher in AG-AG compared to AG-ANT muscles in all three frequency bands, indicating a predilection for functional synergies supporting reciprocal rather than stiffness control. Coherence increased with increasing task difficulty, only in AG-ANT muscles in the low frequency band (0–5 Hz), reflecting subcortical inputs and only in AG-AG group in the high frequency band (16–40 Hz), reflecting corticospinal inputs. Therefore, common neural inputs to both AG-AG and AG-ANT muscles increase with difficulty but are likely driven by different sources of input to spinal alpha motor neurons.

## Introduction

From a neuromuscular perspective, standing balance is maintained through coordinated activation of multiple lower extremity muscles, organized in functional synergies^1–3^. Neural control can be simplified by synchronized or common inputs which activate the muscles comprising a functional synergy as a single unit, instead of separate neural signals to each muscle^3–6^. Biomechanically, in order to maintain balance, the body’s center of mass (COM) dynamics must be appropriately controlled relative to the base of support (BOS) i.e., the contact area between the body and the support surface^7,8^. If the BOS becomes smaller, the COM has to be confined within a smaller area, thereby increasing task difficulty. Such an increase in task difficulty is evidenced by an increase in center of pressure (COP) amplitude and velocity^8,9^. The co-ordination of leg muscle activation is related to COP movements^1,2^ and consequently task difficulty. Therefore, we examined how an increase in task difficulty influences the common inputs which can support the aforementioned co-ordination.

During voluntary or anticipatory COM movements, EMG co-variance shows a reciprocal pattern i.e., groups of anterior or posterior muscles are activated alternately, and not simultaneously^2,3,9^. When task difficulty increases, two or more agonist (AG-AG) muscles may be co-activated to increase torque^3,10,11^, but agonists and antagonists are usually activated separately. Additionally, in some difficult situations like standing on an unstable surface, synergies comprising agonist-antagonist (AG-ANT) muscles can emerge^2^. Co-activation of antagonistic muscles can increase the stiffness of a joint, which in turn can reduce displacement in response to perturbations, without the need for active neural control involving a feedback loop. However, when task difficulty increases, greater stiffness increases the likelihood of losing balance in response to perturbations^12,13^. Therefore, though we expect both AG-AG and AG-ANT common inputs to increase with task difficulty, we expect the strength of common inputs to be greater in AG-AG compared to AG-ANT muscles.

The EMG signal contains spectral information about motor neuron firing and the motor unit action potentials^6,14^. Common presynaptic inputs to the motor neuron pools of two or more muscles can synchronize their firing frequency. The strength of such synchronization becomes apparent in the coherence which is a measure of correlation in the frequency domain, between trains of action potentials discharged by motor neurons innervating two muscles. Therefore, common neural inputs to different muscles can be inferred based on EMG-EMG coherence^15,16^. Neural inputs to muscles at different frequencies are characteristic of activity in different brain areas and circuits. Therefore, the source of the presynaptic common inputs (spinal, subcortical or corticospinal) can be inferred based on the frequencies at which coherences emerge^16^. In standing, coherence has been reported in the 0–5 Hz and 6–15 Hz bands, both of which reflect subcortical inputs though 6–15 Hz may also have some corticospinal contributions^14,17^. As task difficulty increases, corticospinal excitability of leg muscles increases^18–20^, suggesting greater cortical involvement in balance control. Therefore, we hypothesize that in difficult standing tasks, coherence will also emerge at higher frequencies (>15 Hz), reflecting corticospinal inputs^16^.

The primary purpose of this study was to determine how common inputs to AG-AG and AG-ANT muscle pairs, in 3 frequency bands (low: 0–5 Hz, medium: 6–15 Hz and high: 16–40 Hz), change when task difficulty is manipulated by decreasing the BOS in standing. We expect AG-AG coherence to be greater than AG-ANT coherence, and with increasing task difficulty, we hypothesize a larger increase in AG-AG compared to AG-ANT coherence. We expect high frequency coherence reflecting corticospinal inputs to emerge only in the more difficult tasks. Given a lack of previous data, it is premature to predict whether AG-AG and AG-ANT common inputs will be differentially driven by subcortical or corticospinal inputs. Our data will help to clarify how common inputs can simplify the co-ordination of lower extremity muscles in standing. Specifically, we aim to determine whether common neural inputs from cortical and subcortical sources favor reciprocal or stiffness control, using EMG-EMG coherence.

## Methods

### Participants

Twenty healthy young adults (21.0 ± 1.3 y, 9F) without current lower extremity injury, or neurological and orthopedic conditions known to impair standing balance, volunteered for the study. Data were acquired during a single 45 min long lab visit. The Medical Ethical Committee of the University Medical Center Groningen approved the study protocol and informed consent document, and the study was conducted according to the Declaration of Helsinki^21^. We determined foot dominance^22^ using 3 questions about use preference.

### Procedures

Participants completed four tasks in random order, with 2–3 min rest between tasks: (1) wide stance (feet shoulder width apart); (2) narrow stance (feet together); (3) tandem stance (dominant foot posterior), and (4) one leg stance (dominant foot). For each task, participants performed two, 45-s-long trials. Participants wore socks, crossed their arms across the chest and focused their vision on a cross displayed on a projection screen at a distance of ~3 m.

### Data Acquisition

Wireless sensors (dimensions – 37*26*15 mm, electrode material – silver; Trigno™ Wireless System, Delsys, Natick, MA, USA) were used to record EMG from 6 muscles on the dominant side: soleus (Sol); lateral gastrocnemius (LG); tibialis anterior (TA); peroneus longus (PL); biceps femoris (BF), and rectus femoris (RF). The signal was amplified 1000 times and sampled at 5.0 kHz using data acquisition interface and software (Power 1401 and Signal v5.11, Cambridge Electronic Design Ltd, Cambridge UK).

The net ground reaction forces act on a point, within the BOS, called the COP^8^. Dynamics of COP movements provide insight into the neuromuscular control which ensures that COM movements relative to the BOS are controlled in a manner that minimizes the risk of a fall. Therefore, COP data were acquired to confirm whether the BOS limitation did in fact increase task difficulty illustrated by an increase in COP velocity and area. COP location was calculated using moment data obtained from 2 force plates (Bertec 4060-08, Columbus, OH, USA) embedded in the floor, sampled at 200 Hz and acquired using a custom LabVIEW script (v2015, National Instruments, Austin, TX, USA).

### Data Analysis

From each 45 s trial, the middle 30 s of EMG and COP data were selected. The EMG was first visually inspected for any artifacts. Data for one participant was excluded due to noise confirmed by spectral analysis which showed high power across a large range of frequencies. For the rest of the participants, the data were bandpass filtered using a 4^th^ order dual pass Butterworth filter with 20 and 500 Hz cut-offs. A combination of computational and experimental approaches^14,23^ show that motor unit firing information is more easily discernable in rectified data, especially when the motor unit action potentials vary in shape, as is expected in surface EMG. Therefore, we rectified the data using the Hilbert transform because it improves the distinction between the motor unit firing (which we aim to examine) and high frequency voltage fluctuations in the motor unit action potentials^14,24^. Subsequently, the two trials were concatenated to obtain a 60 s long record.

**Figure 1:**
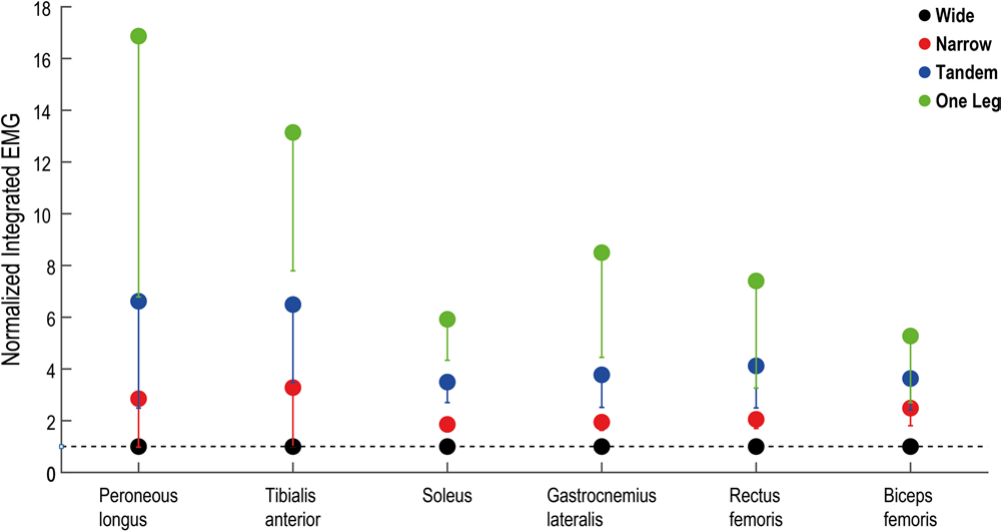
Activation levels of the 6 muscles in the 4 tasks, normalized by activation in wide stance. Activation levels are depicted as EMG integrated over 60 s, and averaged across participants. Error bars depict standard deviations. Horizontal dotted line represents the activation in wide stance.

In order to examine the EMG levels during each task, the concatenated time series for each muscle was integrated (iEMG)^4,5^ and the iEMG in narrow, tandem and one leg stance were normalized by the iEMG in wide stance. Therefore, the levels of activation in the other standing tasks were represented as multiples of activation in wide stance (Fig. 1). Thereafter, the normalized iEMG for 6 muscles were organized in a vector representing the EMG pattern for each task^4,5,25^. To determine whether the EMG patterns in the difficult tasks were similar to wide stance (or not), we estimated the cosine of the angle between the muscle activation vectors for wide stance and the other tasks. High cosine values (close to 1) indicate that a similar EMG pattern is used in the other standing tasks compared to wide stance^4,5,25^. This analysis allowed us to confirm that the relative activation levels of the different muscles were similar across tasks and the selected muscles were relevant for all our tasks. Cross-spectrum (*f*_*xy*_) of pairs of muscles and auto-spectrum (*f*_*xx*_ and *f*_*yy*_) of individual muscles was determined using Welch’s periodogram method. Estimates were obtained using 1 s (5000 data points) long Hanning windows without overlap, resulting in 1 Hz frequency resolution. Intermuscular coherence was estimated by normalizing the squared cross-spectrum by the product of the auto-spectra^26^ –

$${|{R}_{xy}(\lambda )|}^{2}=\frac{{|{f}_{xy}(\lambda )|}^{2}}{{|{f}_{xx}(\lambda ){f}_{yy}(\lambda )|}^{2}}$$

Single-pair coherence was estimated for the following AG-AG pairs: Sol-LG, Sol-PL, LG-PL; and AG-ANT pairs: Sol-TA, LG-TA, PL-TA, RF-BF. Coherence is reported in the 0–55 Hz range and considered to be significant if it exceeds the confidence limit (at α = 0.05) for the number of segments (L) used to estimate the spectrum^27^ –

$$1-{(1-\alpha )}^{\frac{1}{L-1}}$$

TA-PL coherence was significant across all frequencies indicating cross-talk^28^, and was therefore not included in any further analysis. For the remaining pairs, pooled coherence was estimated separately for AG-AG and AG-ANT pairs using the following equation^29^ –

$$\frac{{|\sum _{i=1}^{k}{f}_{xy}(\lambda ){L}_{i}|}^{2}}{\sum _{i=1}^{k}\,{f}_{xx}(\lambda ){L}_{i})\sum _{i=1}^{k}\,{f}_{yy}(\lambda ){L}_{i})}$$

where, k is the number of pairs pooled together (3 each for AG-AG and AG-ANT), and L_i_ is the total number of segments used for estimating the spectrum.

COP data were lowpass filtered using a 4^th^ order dual pass Butterworth filter with 5 Hz cut-off, and the two trials were concatenated. COP velocity (COPvel) and area (COParea) were calculated to characterize COP dynamics in each task.

Coherence data for one participant was excluded due to noisy EMG, and COP data was not available for 2 participants due to technical problems. Therefore, the final coherence analysis included 19 participants and the COP analysis included 17 participants.

### Statistics

Repeated measures ANOVAs were used to test for effect of task difficulty on all the COP outcomes. The COP area data were log transformed since it was not normally distributed. Six repeated measures ANOVAs were used to examine the effect of task difficulty on activation level (iEMG) of each muscle. Both single pair and pooled coherence were Fisher transformed and subsequently integrated in 3 separate frequency bands – 0–5 Hz (low), 6–15 Hz (med) and 16–40 Hz (high). These frequency bands were chosen based on the significant regions observed in our data, previously reported standing data^17,30,31^, and probable neural origin^16,28^ of coherent signals to different muscles. For pooled coherence (expressed as z-score*Hz), separate 2*4 repeated measures ANOVAs were run for each frequency band, to test for main effects of muscle group (AG-AG and ANT-ANT) and task difficulty (wide, narrow, tandem and one leg), and muscle group by task interaction. Since the data were not normally distributed, log transformation was applied before running the ANOVAs. The significance level was set at α = 0.05. Post-hoc paired t-tests were run to examine whether coherence in any of the difficult tasks differed significantly from wide stance. These tests were done separately for the AG-AG and AG-ANT groups leading to 3 pairwise comparisons for each group and Bonferroni adjusted significance level of α = 0.017. Further post-hoc tests were used to examine the difference between AG-AG and AG-ANT group, separately in each task. Since only one comparison was made in each task, correction of the alpha level was not required.

**Figure 2:**
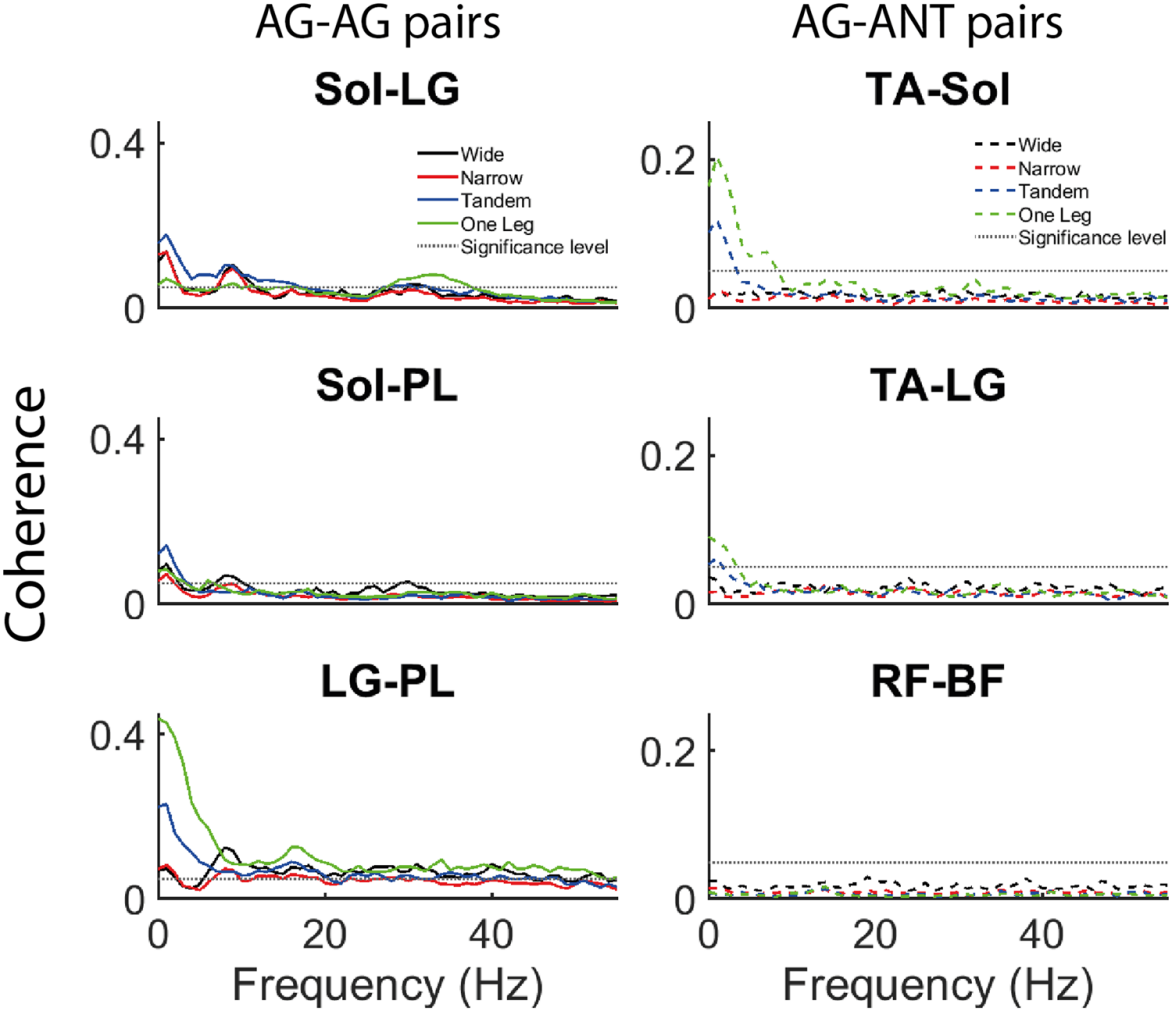
Effect of task difficulty on non-pooled coherence. Left and right panel depict individual muscle pair coherence for AG-AG (Sol-LG, Sol-PL, LG-PL; solid lines) and AG-ANT (Sol-TA, LG-TA, RF-BF; broken lines) pairs respectively. Note different y-axis scales for the left and right panels.

For single-pair coherence, 3*4 repeated measures ANOVAs were run separately for each frequency band and for the AG-AG and AG-ANT pairs. Coherence in none of the AG-ANT pairs exceeded the significance level in the high frequency range. Therefore, no further statistical analyses were run for these data. Post-hoc t-tests were used to compare wide stance with the other tasks separately for each pair and to compare between muscle pairs separately for each task. This allowed us to examine differences between individual muscle pairs within the AG-AG and AG-ANT groups. In both cases, 3 paired t-tests were conducted and the Bonferroni corrected alpha value was 0.017. Post-hoc tests were not computed for comparisons in which the coherence in both tasks or both muscle pairs did not exceed the significance level (see Fig. 2). ANOVA effect sizes were estimated using partial eta squared, with <0.25, 0.26–0.63 and >0.63 considered small, medium and large effect sizes respectively^32,33^. For post-hoc tests, Cohen’s d was used and 0.21–0.50, 0.51–0.79 and >0.79 were considered small, medium and large effect sizes respectively^34^. Coherence values were inverse z-transformed for the figures.

## Results

### Center of pressure

Table 1 shows the main effect of task on COP velocity and area (p < 0.001), which increased with increasing task difficulty.

**Table 1:** Effects of standing task difficulty on center of pressure.

	Wide	Narrow	Tandem	One leg	F(df); p-value	ES
Velocity (cm/s)	1.0 ± 0.2	1.1 ± 0.1	2.2 ± 0.7	2.2 ± 0.4	F_(3,48)_ = 53.48; p = < 0.001*	0.77
Area (cm^2^)	0.5 ± 0.3	1.0 ± 0.6	3.3 ± 1.2	6.6 ± 1.7	F_(3,48)_ = 199.11; p = < 0.001*	0.93

Data presented as mean ± SD; *Significant task main effect, p < 0.05, df – degrees of freedom, ES – effect size (partial eta squared), 0.63 - large^28^.

### EMG activation levels and patterns

The activation level of each muscle (iEMG) increased with increasing task difficulty (p < 0.001; Fig. 1). Additionally, in all tasks the EMG pattern was similar to the pattern in wide stance. Specifically, the cosine of the angles between the muscle activation vectors for wide stance and the other tasks were - narrow (0.94 ± 0.07), tandem (0.93 ± 0.07) and one leg (0.88 ± 0.08).

### Pooled coherence

**Figure 3:**
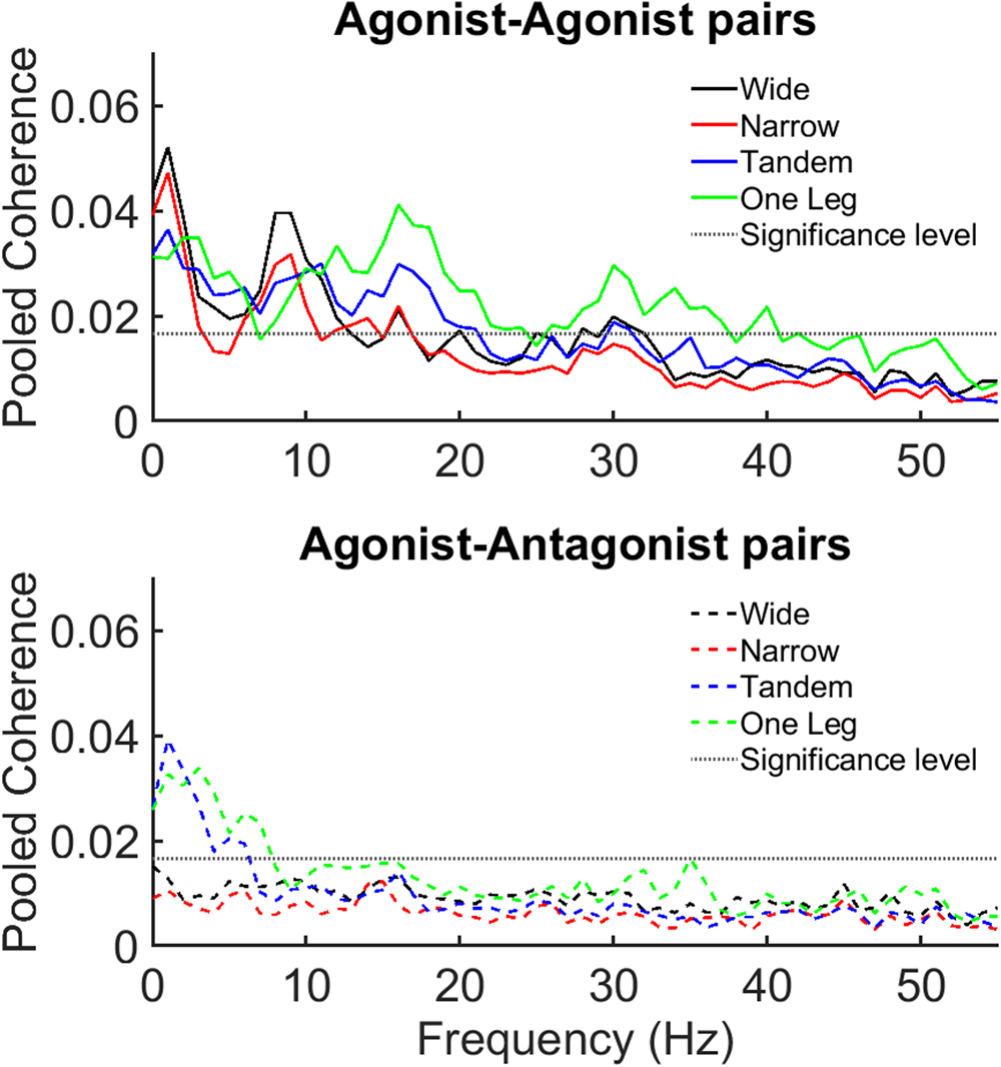
Effect of task difficulty on pooled coherence. Coherence pooled across the AG-AG (Sol-LG, Sol-PL, LG-PL) and AG-ANT (Sol-TA, LG-TA, RF-BF) muscles pairs. Solid lines depict AG-AG muscles and broken lines depict AG-ANT muscles.

**Figure 4:**
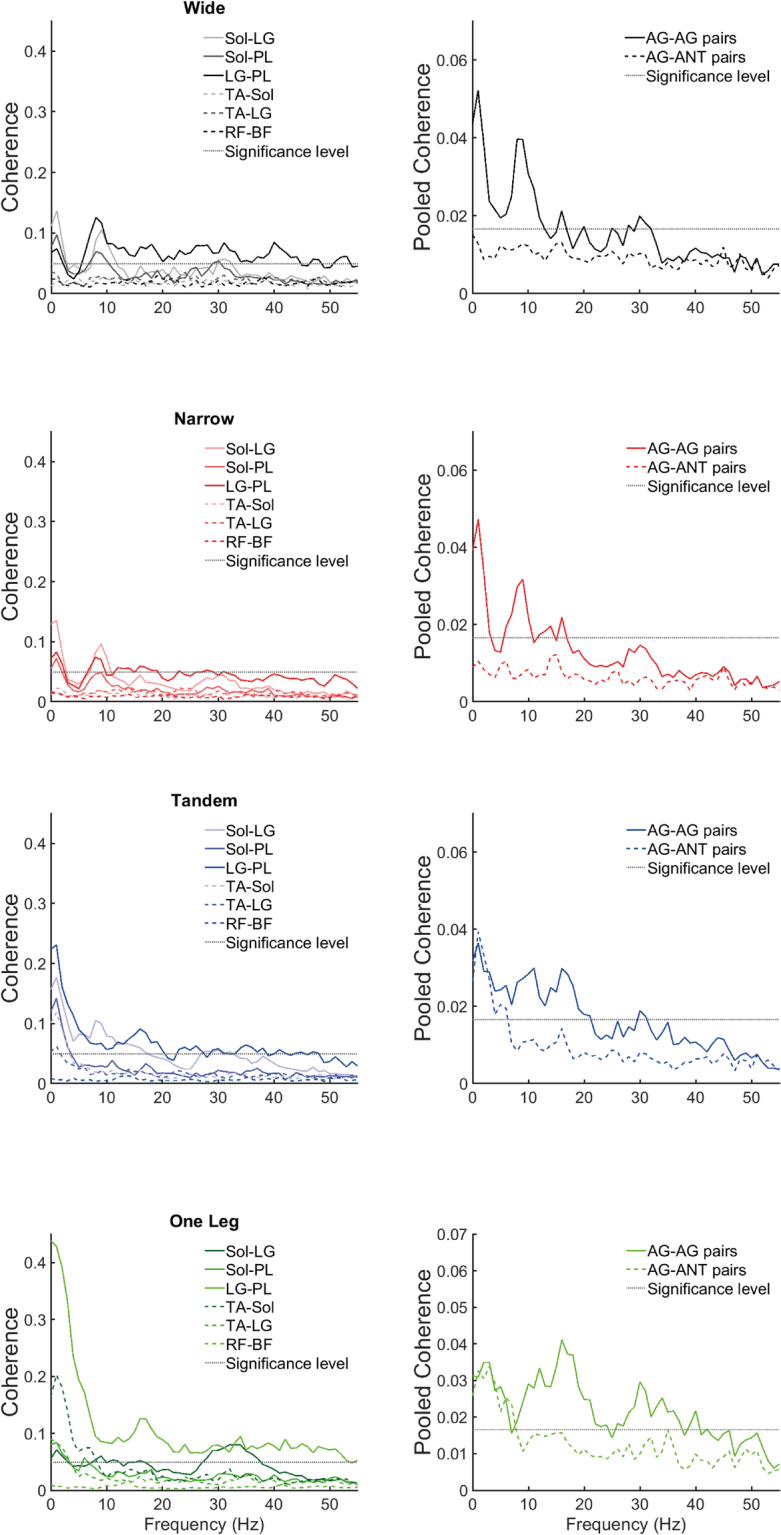
Effect of muscle group (AG-AG or AG-ANT) on coherence. Left panels depict individual muscle pair coherence and right panels show coherence pooled across the AG-AG (Sol-LG, Sol-PL, LG-PL) and AG-ANT (Sol-TA, LG-TA, RF-BF) muscles pairs. Solid lines depict AG-AG muscles and broken lines depict AG-ANT muscles.

**Figure 5:**
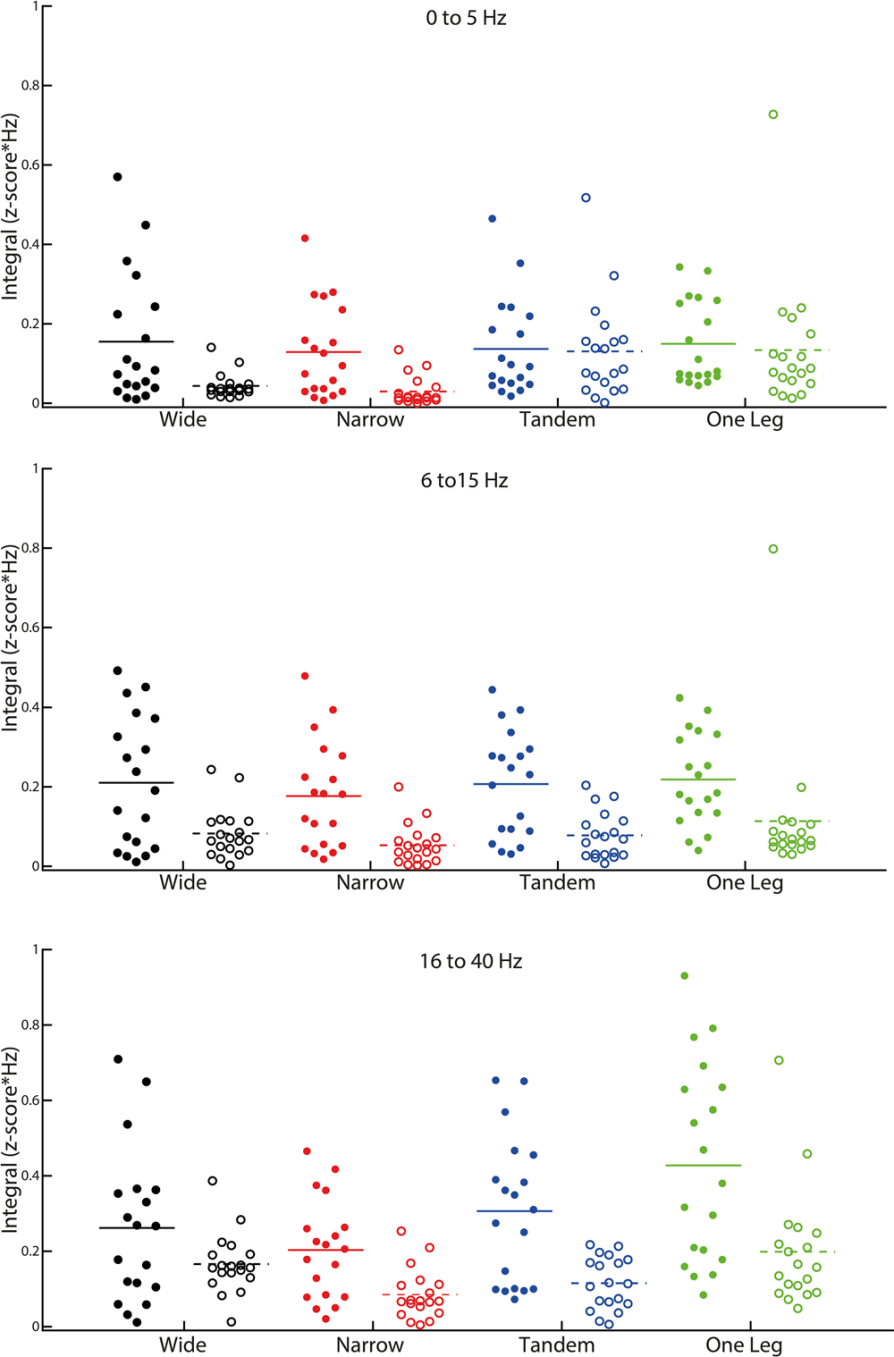
Effects of muscle group, task difficulty, and task difficulty by muscle group interaction on pooled coherence in three frequency bands (0 to 5, 6 to 15, 16 to 50 Hz). Filled circles represent AG-AG coherence and open circles represent AG-ANT coherence. Horizontal lines depict the mean.

In all frequency bands (Figs 2, 3 and 5), coherence was higher in AG-AG compared to AG-ANT group (muscle group main effect). Additionally, in the low and high frequency bands there was a task main effect and an interaction between task difficulty and muscle group (Figs 4 and 5). Post hoc paired t-tests revealed that in the low frequency band, only AG-ANT coherence was higher in one leg compared to wide, t(18) = −3.00, p = 0.008, Cohen’s d = 0.68, mean difference = 0.1 z-score*Hz; and lower in narrow compared to wide t(18) = 2.85, p = 0.011, Cohen’s d = 0.65, mean difference = 0.01 z-score*Hz. Additionally, AG-AG coherence was higher than AG-ANT coherence only in wide (t(18) = 3.41, p = 0.003, Cohen’s d = 0.92, ∆ = 0.88 z-score*Hz) and narrow (t(18) = 3.53, p = 0.002, Cohen’s d = 1.32, ∆ = 1.58 z-score*Hz) stance. In the high frequency band, AG- AG coherence was higher in one leg compared to wide t(18) = −4.19, p = 0.001, Cohen’s d = 0.96, mean difference = 0.24 z-score*Hz; while AG-ANT coherence was lower in narrow compared to wide t(18) = 3.82, p = 0.001, Cohen’s d = 0.88, mean difference = 0.08 z-score*Hz. Additionally, AG-AG coherence was higher than AG-ANT coherence in narrow (t(18) = 3.74, p = 0.001, Cohen’s d = 1.05, ∆ = 0.98 z-score*Hz), tandem (t(18) = 4.97, p < 0.001, Cohen’s d = 1.21, ∆ = 1.08 z-score*Hz) and one leg (t(18) = 4.80, p < 0.001, Cohen’s d = 1.12, ∆ = 0.86 z-score*Hz) stance. Note that in both the low and high frequency bands, the lower coherence in narrow compared to wide stance is statistically significant, but the mean differences are much smaller than the increase from wide to one leg stance and cannot be meaningfully interpreted. In the medium frequency band, there was a main effect of task but a relatively small effect size. Additionally, none of the post-hoc tests were significant indicating that coherence in any of the difficult tasks did not differ from the control task i.e., wide stance. Table 2 shows the p-values, F values, degrees of freedom and effect sizes (partial eta squared) for the ANOVAs.

**Table 2:** Effects of muscle group (AG-AG or AG-ANT) and task difficulty on pooled coherence (integrated in 3 frequency bands).

	Muscle group M*ain effect*		Task difficulty *Main effect*		Task difficulty* Muscle group *Interaction*	
	F(df); p-value	ES	F(df); p-value	ES	F(df); p-value	ES
Low (0–5 Hz)	F_(1,18)_ = 14.05; p = < 0.001*	0.44	F_(3,54)_ = 9.01; p = < 0.001*	0.33	F_(3,54)_ = 5.03; p = 0.004*	0.22
Medium (6–15 Hz)	F_(1,18)_ = 27.20; p = < 0.001*	0.60	F_(3,54)_ = 3.90; p = 0.014*	0.18	F_(3,54)_ = 1.95; p = 0.13	0.10
High (16–40 Hz)	F_(1,18)_ = 22.64; p = < 0.001*	0.56	F_(3,54)_ = 11.68; p = < 0.001*	0.39	F_(3,54)_ = 5.94; p = < 0.001*	0.25

*Significant at p < 0.05.

AG-AG group: Sol-LG, Sol-PL, LG-PL.

AG-ANT group: Sol-TA, LG-TA, RF-BF.

df – degrees of freedom.

ES – effect size (partial eta squared), <0.25 – small and 0.26–0.63 - medium^28^.

#### Non-pooled coherence

In the AG-AG group, the main effect of task, main effect of muscle pair and the interaction were significant in all 3 frequency bands (Table 3). In the AG-ANT group, the main effect of task, main effect of muscle pair and the interaction were significant in the low and medium frequency bands (Table 4). In some tasks, the coherence for some muscle pairs did not exceed the significance level (see Fig. 2). Post-hoc tests were not computed if coherence in both pairs or tasks did not exceed the significance level.

**Table 3:** Agonist-agoinst (AG-AG) group - effects of muscle pair and task difficulty on non-pooled coherence (integrated in 3 frequency bands).

AG-AG	Muscle pair Main effect			Task difficulty *Main effect*			Task difficulty* Muscle pair *Interaction*		
	*F(3,36)*	*p*	*ES*	*F(3,54)*	*p*	*ES*	*F(6,108)*	*p*	*ES*
*Low* *(0–5* *Hz)*	8.29	0.001*	0.32	13.93	<0.001*	0.44	9.54	<0.001*	0.35
*Medium* *(6–15* *Hz)*	10.59	<0.001*	0.37	3.84	0.015*	0.18	3.71	0.002*	0.17
*High* *(16–40* *Hz)*	15.82	<0.001*	0.47	9.88	<0.001*	0.35	3.16	0.007*	0.15

*Significant at p < 0.05.

AG-AG group: Sol-LG, Sol-PL, LG-PL.

ES – effect size (partial eta squared), <0.25 – small and 0.26–0.63 - medium^14^.

**Table 4:** Agonist-antagoinst (AG-ANT) group - effects of muscle pair and task difficulty on non-pooled coherence (integrated in 3 frequency bands).

AG-ANT	Muscle pair *Main effect*			Task difficulty *Main effect*			Task difficulty* Muscle pair *Interaction*		
	*F(3,36)*	*p*	*ES*	*F(3,54)*	*p*	*ES*	*F(6,108)*	*p*	*ES*
*Low* *(0–5* *Hz)*	58.34	<0.001*	0.76	17.83	<0.001*	0.50	21.12	<0.001*	0.54
*Medium* *(6–15* *Hz)*	11.99	<0.001*	0.40	6.56	0.001*	0.27	6.35	<0.001*	0.26
*High* *(16–40* *Hz)*	*Not tested because coherence for none of the pairs exceeded significance level*								

*Significant at p < 0.05.

AG-ANT group: Sol-TA, LG-TA, RF-BF.

ES – effect size (partial eta squared), <0.25 – small and 0.26–0.63 - medium^14^.

Post-hoc tests for the differences between individual muscle pairs, in each task are shown Tables 5 and 6. In the AG-AG group, in tandem and one leg stance, LG-PL coherence was higher than the other 2 pairs, and Sol-LG coherence was higher than Sol-PL. In the AG-ANT group, in tandem and one leg stance, TA-Sol and TA-LG coherence was higher than RF-BF coherence.

**Table 5:** Agonist-agoinst (AG-AG) group – post-hoc tests comparing non-pooled coherence between the different muscle pairs (integrated in 3 frequency bands).

		Wide			Narrow			Tandem			One Leg		
		t(18)	p	ES	t(18)	p	ES	t(18)	p	ES	t(18)	p	ES
LG-PL > Sol-LG	0–5 Hz	1.42	0.17	0.28	1.40	0.18	0.31	−0.85	0.41	0.31	**−9.12**	<0.001*	**2.73**
	6–15 Hz	−1.71	0.10	0.41	0.27	0.79	0.09	0.13	0.90	0.03	**−4.56**	<0.001*	**1.30**
	16–40 Hz	−1.86	0.80	0.51	−0.58	0.57	0.20	−1.58	0.13	0.34	−2.09	0.05	0.43
LG-PL > Sol-PL	0–5 Hz	0.55	0.59	0.13	−0.70	0.49	0.22	−1.34	0.20	0.42	**−11.03**	<0.001*	**2.61**
	6–15 Hz	−2.41	0.03	0.68	−2.34	0.04	0.63	**−4.75**	<0.001*	**1.20**	**−7.71**	<0.001*	**1.96**
	16–40 Hz	−2.48	0.02	0.64	**−3.02**	0.007*	**0.73**	**−6.02**	<0.001*	**1.46**	**−5.07**	<0.001*	**1.34**
Sol-LG > Sol-PL	0–5 Hz	0.56	0.58	0.15	1.77	0.1	0.50	0.51	0.62	0.11	−0.91	0.37	0.19
	6–15 Hz	0.81	0.43	0.26	2.26	0.04	0.73	**3.42**	**0.003**	**1.11**	1.27	0.22	0.43
	16–40 Hz	Not tested						**3.45**	0.003*	**1.07**	**4.08**	0.001*	**1.03**

Post-hoc tests not computed if coherence for both pairs did not exceed the significance level (see Fig. 2).

*Significant at p < 0.017 (Bonferroni corrected).

ES - effect size (Cohen’s d), 0.21–0.50 – small, 0.51–0.79 - medium and >0.79 – large^15^.

**Table 6:** Agonist-antagoinst (AG-ANT) group – post-hoc tests comparing non-pooled coherence between the different muscle pairs (integrated in 3 frequency bands).

		Wide			Narrow			Tandem			One Leg		
		t(18)	p	ES	t(18)	p	ES	t(18)	p	ES	t(18)	p	ES
TA-Sol = TA-LG	0–5 Hz	−0.14	0.89	0.04	1.03	0.32	0.20	2.19	0.04	0.44	2.47	0.02	0.74
	6–15 Hz	Not tested									1.47	0.16	0.37
TA-Sol > RF-BF	0–5 Hz	0.50	0.62	0.15	0.06	0.95	0.01	**6.07**	<0.001*	**1.95**	**12.51**	<0.001*	**3.10**
	6–15 Hz	Not tested									**4.59**	<0.001*	**1.62**
TA-LG > RF-BF	0–5 Hz	0.57	0.57	0.18	−0.65	0.52	0.23	**4.89**	<0.001*	**1.69**	**9.40**	<0.001*	**3.07**
	6–15 Hz	Not tested											

Post-hoc tests not computed if coherence for both pairs did not exceed the significance level (see Fig. 2).

*Significant at p < 0.017 (Bonferroni corrected).

ES - effect size (Cohen’s d), 0.21–0.50 – small, 0.51–0.79 - medium and >0.79 – large^15^.

Post-hoc tests for the differences between tasks, for each individual pair are shown in Tables 7 and 8. In the AG-AG group, LG-PL coherence was higher in one leg and tandem, compared to wide stance. Also, Sol-PL coherence was lower in narrow and tandem, compared to wide stance possibly due to a peak observed in wide stance at approximately 10 Hz.

**Table 7:** Agonist-agoinst (AG-AG) group – post-hoc tests comparing non-pooled coherence between the different tasks (integrated in 3 frequency bands).

		Sol-LG			Sol-PL			LG-PL		
		t(18)	p	ES	t(18)	p	ES	t(18)	p	ES
Narrow **<**Wide	0–5 Hz	−0.01	0.99	0	1.60	0.13	0.40	−2.51	0.02	0.74
	6–15 Hz	0.76	0.46	0.11	**3.37**	0.003*	**0.62**	2.19	0.04	0.58
	16–40 Hz	Not tested			Not tested			2.48	0.02	0.55
Tandem >Wide^1^ Tandem <Wide^2^	0–5 Hz	−1.12	0.28	0.24	−0.85	0.41	0.29	**−2.62**	0.017*	**0.86** ^1^
	6–15 Hz	−0.86	0.40	0.18	**2.65**	0.016*	**0.81** ^2^	0.78	0.45	0.25
	16–40 Hz	−0.45	0.66	0.10	Not tested			0.08	0.94	0.02
One Leg >Wide	0–5 Hz	0.96	0.35	0.21	−0.42	0.68	0.13	**−10.16**	<0.001*	**3.00**
	6–15 Hz	0.63	0.54	0.18	1.04	0.31	0.32	−1.58	0.13	0.47
	16–40 Hz	−1.91	0.07	0.38	Not tested			−1.19	0.15	0.25

Post-hoc tests not computed if coherence in both tasks did not exceed the significance level (see Fig. 2).

*Significant at p < 0.017 (Bonferroni corrected).

ES - effect size (Cohen’s d), 0.21–0.50 – small, 0.51–0.79 - medium and >0.79 – large^15^.

**Table 8:** Agonist-antagoinst (AG-AG) group – post-hoc tests comparing non-pooled coherence between wide stance and each of the other tasks (integrated in 3 frequency bands).

		TA-Sol			TA-LG			RF-BF		
		t(18)	p	ES	t(18)	p	ES	t(18)	p	ES
Narrow = Wide	0–5 Hz	Not tested						Not tested		
	6–15 Hz									
Tandem >Wide	0–5 Hz	**−2.91**	0.009*	**0.92**	−1.32	0.20	0.42			
	6–15 Hz	Not tested								
One Leg >Wide	0–5 Hz	**−6.82**	<0.001*	**2.14**	**−5.03**	<0.001*	**1.83**			
	6–15 Hz	−1.04	0.31	0.35	Not tested					

Post-hoc tests not computed if coherence in both tasks did not exceed the significance level (see Fig. 2).

*Significant at p < 0.017 (Bonferroni corrected).

ES - effect size (Cohen’s d), 0.21–0.50 – small, 0.51–0.79 - medium and >0.79 – large^15^.

## Discussion

We examined the effects of task difficulty on common neural inputs to lower extremity AG-AG and AG-ANT muscles in standing, in healthy young adults. The increase in COP velocity and area confirmed that the experimental manipulations increased task difficulty. In agreement with the hypothesis, we found higher coherence in AG-AG compared to AG-ANT pairs in the three frequency bands. Coherence in the difficult one leg task was higher than wide stance, only in the AG-ANT group in the low frequency band (0–5 Hz), reflecting common subcortical inputs, and only in AG-AG group in the high frequency band (16–40 Hz), reflecting common corticospinal inputs. Therefore, common neural inputs to both AG-AG and AG-ANT muscles increase with difficulty but are likely driven by different sources of input to spinal alpha motor neurons. Our data supports the argument that common neural inputs to groups of muscles simplify the co-ordination of lower extremity muscles to control standing balance. Biomechanically, we expected the ankle muscles to be more important for maintaining balance in our experimental tasks. We included some knee muscles because previous muscle synergy analyses showed that knee muscles are also important for COP control in standing^1,2^. However, RF-BF coherence was consistently low, possibly because these muscles are not as functionally relevant as the ankle muscles for the chosen tasks. Therefore, our conclusions regarding AG-ANT common input are limited to the ankle muscles. Further studies are required to determine if other groups/pairs of knee muscles receive common inputs in standing tasks.

Common inputs to alpha motor neurons can arise from supraspinal inputs, afferent feedback or spinal connections between motor neuron pools^31^. Even though the exact neurophysiological origin of 0–5 coherence between unilateral muscles is not known, it is maintained in patients with cortical and capsular strokes^35,36^, suggesting a subcortical source^16^. Coherence in the 6–15 Hz band may have contributions from both cortical and subcortical sources and there is some evidence that it arises from neural networks comprising the cerebellum, sensorimotor cortex, inferior olive and thalamus^16,37,38^. In the 16–40 Hz range, EMG-EMG coherence is diminished in spinal cord injury patients^28^ and EMG is coherent with cortical activity recorded using EEG or MEG^37,39^, providing strong evidence for a corticospinal origin. A limitation of this method is that increase in EMG- EMG coherence cannot be directly interpreted as an increase in the level of co-activation as quantified using EMG amplitude. High EMG-EMG coherence indicates that motor units in both muscles receive neural inputs at the same frequencies^14^. Though such neural inputs may not necessarily arrive at both muscles at the same time, increase in AG-ANT coherence does suggest that the neural inputs can support a co-ordination pattern that increases stiffness. Given this physiological background, in the following sections we discuss the relevance of common neural inputs for the coordination of lower extremity muscles in standing. In agreement with the hypothesis, we found that AG-AG coherence which supports direction specific reciprocal muscle control is usually higher that AG-ANT coherence which supports stiffness control. However, low frequency coherence reflecting subcortical common inputs were almost equivalent in the AG-AG and AG-ANT groups in the two most difficult tasks (Fig. 5). This finding must be interpreted in conjunction with the observations in other frequency bands. In both the medium and high frequency bands AG-AG coherence is consistently higher than AG-ANT coherence. It is thus clear that there is a bias towards functional synergies which can create direction specific torques to counteract gravitational torques. However, when there is a need to increase AG-ANT co-activation, it is likely supported by sub-cortical inputs to alpha motor neurons. On the other hand, task related increases in AG-AG coherence are presumably driven primarily by corticospinal inputs.

In standing, 0–5 Hz coherence is observed between bilateral homologous muscles^17,31,40^ and unilateral muscles acting on different joints^4,5,41^ and our study adds to the limited evidence for 0–5 Hz coherence between unilateral muscles acting at the same joint^17^. We found no effect of task difficulty on AG-AG coherence pooled across three pairs, but in agreement with previous data^30^ we found that pooled AG-ANT coherence increases when task difficulty increases due to reductions in BOS. The examination of individual muscle pairs (i.e., non-pooled coherence) supports the findings from pooled data in general. However, some further nuances become apparent. Compared to other AG-AG pairs, LG-PL coherence is relatively higher while Sol-PL coherence is relatively low, especially in the difficult tasks (Table 5). Additionally, task difficulty related increase in coherence is seen only in the LG-PL pair. Though Sol and PL are both plantarflexors, Sol is an invertor and PL is an evertor, making them antagonists in the mediolateral (ML) direction. On the other hand, LG and PL are agonists in both anteroposterior (AP) and ML directions. These findings are in line with a previous study^10^ which examined coherence between different pairs of plantarflexors in standing and found the highest coherence between medial gactrocnemius (MG) and soleus, which are both invertors. Also, in the 0–5 Hz range, lower extremity EMG is coherent with COP movements^10,42^ suggesting that inputs to particular pairs of muscle may be synchronized based on the direction of the torques they produce. In other words, coherence between specific muscle pairs may be related to the direction in which their activation shifts the COP. Indeed, LG or PL activation shifts the center of pressure (COP) medially, while MG or SL shift the COP laterally^10,43^. The stronger coherence between LG-PL and MG-Sol in difficult tasks provides evidence for task specific evertor/invertor synergies, which are not required for counteracting the smaller gravitational torques in wide and narrow stance. Similarly, the increase in AG-ANT coherence is driven by TA and Sol which are both invertors. Therefore, our findings support the argument that functional synergies, formed through common neural inputs to different muscles, are specific to the biomechanical demands of the task. A limitation of the present and previous studies is that surface EMG may not accurately reflect motor unit coherence at low frequencies (<5 Hz) and high contraction intensities, as suggested by recent experimental data^44^. However, since surface EMG underestimates low frequency common inputs, task difficulty related effects may in fact be more prominent if intra-muscular recordings are used.

In our data, 6–15 Hz AG-AG coherence is apparent in all the tasks, while there is little or no AG-ANT coherence in any task. Also, there is no effect of task difficulty on either AG-AG or AG-ANT coherence. However, we do observe a peak in AG-AG coherence at ~10 Hz in wide and narrow stance, but not in tandem and one leg stance. Coherence in the 8–12 Hz (with a peak at ~10 Hz) range has previously been reported between bilateral homologous muscles^40,45^. Though we tested coherence between unilateral muscles, the peak in EMG power at 10 Hz is present in the two tasks which require symmetrical activity in both legs. However, it disappears in tandem and one leg stance when the two legs have different biomechanical configurations and consequently muscle activations. Indeed, analysis of non-pooled coherence shows that Sol-PL coherence is lower in tandem, compared to wide stance. Sol-PL coherence is also lower in narrow compared to wide. The 10 Hz peak is also apparent in narrow stance (Fig. 2), although it is smaller than the peak in wide stance. However, visual inspection of the graph (Fig. 2) shows that the peak is present in narrow stance also, although it is smaller than the peak in wide stance. Therefore, we hypothesize that 10 Hz coherence reflects synchronization of muscle activation between the legs. Obata *et al*. found a small 10 Hz peak in coherence between unilateral MG-Sol, but only when vision was occluded in bipedal stance^17^. In our data, the peak is apparent in all three AG-AG pairs and therefore the conflicting findings cannot be attributed to the specific muscle pair. They pooled data across all the participants and used EMG normalized to unit variance, possibly accounting for the differences. Also, it must be noted that 10 Hz oscillations are widespread in the neuromotor system and likely have a multifactorial origin^46^. Further work is required to clarify the reasons for differences in the peaks between tasks, but this observation further emphasizes the biomechanical task specificity of functional synergies.

In standing, high frequency AG-AG coherence becomes apparent only when task difficulty and consequently muscle activation increases. This finding is in line with previous reports examining the effects of BOS manipulations and leaning tasks on coherent inputs to lower extremity muscles^10,47^. It is also in agreement with previous TMS and EEG studies which demonstrate increasing cortical involvement in standing balance control as task difficulty increases^18,19,48–50^. However, a caveat must be added. Currently available measurement techniques allow easier recording of cortical compared to subcortical activity. Since the M1 receives inputs from multiple brain areas, including prefrontal areas, cerebellum and basal ganglia, our findings (and those of TMS and EEG studies) do not rule out the possibility that task-related changes observed in M1 activity are in fact driven by inputs to M1 from other brain areas. Further studies are required to determine if other brain areas drive the synchronization of M1 outputs.

High frequency AG-ANT coherence was not present in any task. Individual motor neurons within the primary motor cortex (M1) may activate multiple AG-AG muscles^51^, possibly though branched descending inputs to spinal motor neuron pools innervating different muscles^6^. Additionally, M1 neurons show strong directional tuning, i.e., they are activated only during movements in one direction^51^. In fact, some M1 neurons also have a inhibitory effects on antagonistic movements^52^. Therefore, the properties of descending inputs from individual M1 neurons to multiple muscles favor coherent AG-AG activation. However, the possibility of synchronized activation of multiple M1 neurons, with differing directional tunings, cannot be ruled out. Additionally, common corticospinal inputs may also arise from other areas like the premotor and supplementary motor areas. Further studies are required to determine whether AG-ANT coherence driven by corticospinal inputs may emerge in other types of tasks and movements. In summary, we demonstrated that common neural input is a likely mechanism underlying the task-specific coordination of lower extremity muscles in standing. This argument is strengthened by the observation that the pattern of coherence reflects the biomechanical demands of each task. Additionally, AG-AG synchronization can be driven by both cortical and subcortical inputs, but when task difficulty increases, corticospinal involvement increases. Conversely, task related changes in AG-ANT synchronization are driven mainly by subcortical inputs.

## Supplementary information

Supplementary Dataset 1

## Data Availability

Data generated or analysed during this study are included in the Supplementary Information files of this article.
